# The relationship between aesthetic preferences of people for ceramic tile design and neural responses: An event-related potential study

**DOI:** 10.3389/fnhum.2022.994195

**Published:** 2022-11-18

**Authors:** Jiayin Chen, Yue Cheng

**Affiliations:** ^1^School of Design and Art, Jingdezhen Ceramic Institute, Jingdezhen, China; ^2^School of Ceramic Art, Jiangxi Arts and Ceramics Technology Institute, Jingdezhen, China

**Keywords:** event-related potentials, aesthetic preferences, ceramic tiles, N100, P200, LPP

## Abstract

**Introduction:**

The aesthetic preferences of people can determine the success of a design and are often closely related to design features. The discovery of designs that match user preferences can provide a reference for designers. Ceramic tiles are widely used in environmental design; however, little attention has been paid to the aesthetic preferences of people for tiles. This study aimed to explore the relationship between aesthetic preferences for tile design and neural responses.

**Materials and methods:**

In this study, two groups of tiles with different preference levels were randomly presented to 16 participants, and their electroencephalograms were recorded. The mean amplitudes of event-related potentials were analyzed by ANOVA.

**Results:**

The results showed that: (1) the aesthetic preferences of people for tiles could modulate brain activity; (2) tiles that people liked triggered higher N100 amplitudes; and (3) tiles that people disliked triggered higher P200 and late positive potential (LPP) amplitudes.

**Discussion:**

These results suggest that N100, P200, and LPP are significantly related to the aesthetic preferences of people for ceramic tiles. The difference in N100 and P200 amplitudes indicates that participants developed aesthetic perceptions of the tiles in the early and middle stages of vision and formed different attention allocations to tiles with varying levels of aesthetic preference; in the middle and late stages of visual processing, the difference in the LPP amplitude indicates that the impression of people for tiles is further deepened in the later stage, forming a top-down emotion-driven evaluation. Exploring the relationship between the aesthetic preferences of people and neural responses is significant in establishing objective aesthetic judgment indicators for tiles and understanding the process of aesthetic cognition. This study provides relevant information for quantitative aesthetic assessments of environmental design, interior design, and marketing involving ceramic tiles.

## Introduction

Ceramic tile is a building material with a long history, representing an exciting fusion between industry, art, and architecture. Ceramic tile only exists as the “skin” of architecture; nevertheless, it can transform the visual artistic effect of the environment (Zhang, 2008). Therefore, tiles designs are gradually becoming more diversified and creative. In addition to homes, ceramic tiles are often used on the interior and exterior of museums, libraries, schools, transit hubs, and other buildings (Zhang, 2016). Compared with most environmental design elements, ceramic tiles are used in many applications. For example, floor-laying materials in China are mainly ceramic tiles, with an area of 8.474 billion square meters, accounting for 71.4% of the total area (China Building Sanitary Ceramics Association, 2021). Thus, it can be seen that ceramic tile has a universal application in environmental design. In addition, many residents living in cities and towns buy ceramic tiles from markets and websites to decorate their houses.

As a type of industrial product design, tiles shape our living environment through various design features such as patterns, textures, and colors (Albors-Garrigós et al., 2009). People often perceive the features of tiles through visual and tactile senses, with the visual sense being the dominant one (Artacho et al., 2020). In terms of visual form, ceramic tiles are associated with art and beauty because of their different color and texture features (Stewart, 1999). Therefore, the form of the tile floor has a significant impact on people’s aesthetic experiences (Agost and Vergara, 2014). Sensory interaction between people and tiles during the purchase will generate an aesthetic experience, and assessing the perception of people seeing ceramic tile products can help provide greater aesthetic pleasure (Artacho et al., 2020). In terms of interior design, many studies have confirmed that interior design elements impact the physical and mental health of people (Evans, 2003; Ghamari and Amor, 2016). For example, Ulrich (1984) found that proper interior window design positively influences the physical and mental health of patients. Mahmood and Tayib (2021) found through questionnaires that using diverse wall colors, beautiful tiles, and durable wall coatings in interior design could improve the psychological comfort of the user. Serra et al. (2021) found that the psychological satisfaction of people with the environment was significantly enhanced when the colors of walls and floors were lighter shades or similar chroma. However, no study has been found on the relationship between the aesthetic preference of tile design and neural responses. Therefore, it is worth exploring the aesthetic preferences of ceramic tiles.

The aesthetic experience of objects is a part of daily life (Wang et al., 2012). The search for causes, mechanisms, and effects of aesthetics has been the focus of philosophical and non-philosophical thinking (Tommaso et al., 2008). The objectivist view of aesthetic theory, which dates back to Plato, states that beauty is the property of an object that produces pleasurable experiences in any suitable perceiver (Feagin and Maynard, 1997; Tatarkiewicz, 2006). The original model theory suggests that aesthetic experience is closely related to the external object that elicits aesthetic experience and spans from very positive to very negative experiences (Palmer et al., 2013). The pleasant experience is closely related to aesthetic preferences and usually occurs when observing and evaluating objects (Brown et al., 2011). Aesthetic preferences refer to how much people appreciate a particular visual stimulus; evaluate the beauty of a product, or how much they like a product (Roberts, 2007). Many studies have proven that aesthetic preferences for design are positively influenced by aesthetic features (Yamamoto and Lambert, 1994; Shieh and Yang, 2008). In addition, aesthetic preferences are essential to the marketing of product design (Baxter, 1995; Sevilla and Townsend, 2016). Therefore, the aesthetic perception of the design can be leveraged to improve the desirability of a product. Regarding theory, Kansei Engineering is a branch of cognitive ergonomics introduced in 1970 that has been applied to various product designs (Nagamachi, 2002). Kansei engineering connects product design features with human emotional preferences and transforms the emotions of the customers into emotional words that communicate product design features (Yang, 2011).

In terms of the subjective measurement of aesthetic preferences, most previous studies used painting and landscape design as stimuli to measure subjective feelings (Beudt and Jacobsen, 2015). The research content mainly focused on the influence of factors such as typicality, complexity, novelty, and symmetry of design and sex or personality on aesthetic preference (Cox and Cox, 2002; Hekkert et al., 2003; Creusen et al., 2010; Forsythe et al., 2011; Cleridou and Furnham, 2014; Hsiu-Feng, 2014). In recent years, some studies on the aesthetic preferences of designs have begun to use more specific designs as stimuli to measure how people feel about the aesthetic qualities of different designs (Nasar, 1994; Leder and Carbon, 2005). For example, Wang et al. studied pendant designs with different aesthetic qualities (ugly/dislike and beauty/like). They found that the diverse preferences of people for pendant designs could produce differences in subjective feelings and physiological reactions (Wang et al., 2012). Furthermore, Ma et al. (2015) conducted a study on the aesthetic preference of architectural design and found that buildings designed by famous designers usually have a higher design aesthetic quality. Research on aesthetic preferences has gradually materialized and is closely related to aesthetic quality. Therefore, this study explores aesthetic preferences for ceramic tiles from the perspective of aesthetic quality.

Previous studies have demonstrated that choosing preferred tiles can effectively increase the satisfaction of participants with their environment. For example, Serrano et al. (2013) used virtual reality technology to allow participants to decorate their rooms with their chosen tiles, and increase their satisfaction with the environment. Agost and Vergara (2014) conducted a questionnaire study and found that light-toned tile floorings increased the preference for the environment. Questionnaires are a commonly used quantitative research tool. However, the feelings that people fill in questionnaires may differ from their real experiences. In addition to the research method, the experimental setting can affect the accuracy of the results. Many previous experimental studies have been conducted in realistic environments (Artacho et al., 2020); however, distractions (e.g., sound and furniture) in realistic environments affect the perception of people seeing tiles.

Some studies have suggested that studying aesthetic preferences using subjective and objective methods may be more accurate and stable (Nayak and Karmakar, 2019). In recent years, neuroaesthetics has established a physiological and methodological basis for studying aesthetic preferences (Chatterjee, 2011; Nadal and Pearce, 2011). Specifically, researchers of neuroaesthetics typically study how aesthetic experiences occur in real time in the brain, and these studies rely on observations that link brain activity to aesthetic experiences. Several regions of the emotional assessment system in the brain contribute to aesthetic experience, including the orbitofrontal and medial frontal cortex, ventral striatum, anterior cingulate gyrus, and insula (Chatterjee and Vartanian, 2014). In addition, many neuroaesthetics studies have found that some common stimuli (such as signs, pictures, and geometric figures) can trigger human implicit human aesthetic preferences when no evaluation or decision-making guidelines are present (Bargh and Ferguson, 2000; Höfel and Jacobsen, 2007; Handy et al., 2010). Given the importance of neuroaesthetic research in neurodesign and neuromarketing (Ma et al., 2008), it is worth considering using neuroaesthetics methods to evaluate the perceptions of people regarding design. In addition, using accurate and immediate neurophysiological techniques to conduct experiments can effectively avoid interference from other factors (Ding et al., 2016; Zhang, 2020). Exploring the relationship between aesthetic preferences and neural responses in tile design will further advance environmental design and neuroaesthetics.

Event-related potential technology (ERP) offers the benefit of high temporal resolution and non-invasive measurement, collecting immediate responses from the human brain and providing information that traditional research methods (e.g., interviews, questionnaires, focus groups, etc.) cannot supply (Hou and Yang, 2020; Edwards and Trujillo, 2021). In addition, previous studies have found that products with different aesthetic preferences generate changes in ERP amplitudes (Ma et al., 2015; Lin et al., 2018). These findings were derived from studies conducted on ERP components. The ERP components can reflect human emotional activity and help people understand the complexities of cognitive function within the brain (Delorme and Makeig, 2004; Folstein and Van Petten, 2008; Lopez-Calderon and Luck, 2014). N100, P200, and LPP are common components of event-related potentials.

Aesthetic preferences in the affect-based evaluation produce different attention allocations and affect ERP components. Aesthetic preference formation involves various cognitive and affective components (Darden and Babin, 1994; Bechara et al., 2000; Silvia and Warburton, 2006). In addition to reward-based valuation (Kawasaki and Yamaguchi, 2012), there is an affect-based appraisal (Handy et al., 2010). The attractiveness of the physical attributes of the stimuli will trigger the appreciation and emotions of pleasantness or unpleasantness, influencing preference evaluation (Handy et al., 2010). In this type of evaluation, people prefer a pleasant design to an unpleasant design (Bechara et al., 2000). Many studies support the view that pleasant emotions brought about by the appearance of a design influence preference judgment. For example, Guo et al. (2016) found that the pleasant appearance of smartphones affects the preference of people. Wang et al. (2021) found that the aesthetics of web interface design influences preference judgment and ERP amplitudes. Our study aims to understand the preferences of people for the appearance of tile design and excluded price factors; therefore, this research is based on the affect-based evaluation.

The N100, P200, and LPP components were confirmed to be related to emotional perception and attention allocation concerning preference assessment.

In the studies of perceptual processing, many researchers have found that the N100 component (which peaks 100–200 ms after stimulation) is closely related to the allocation of attentional resources (Luck et al., 2000; Vogel and Luck, 2000). Keil et al. (2002) found that pleasant and unpleasant emotional stimuli elicited a greater N100 than neutral emotional stimuli. In studies of visual aesthetic preference, many researchers have found that preferred stimulus pictures elicit greater N100 amplitudes (Liu et al., 2021; Guo et al., 2022). Thus, the N100 may be used to reveal the attention allocation related to preference formation (Guo et al., 2018; Liu et al., 2019). However, this is the first study of aesthetic preferences using ERP techniques to analyze aesthetic preferences for tiles. The different experimental stimuli prevented us from concluding whether the aesthetic preferences for tiles could moderate the N100. Therefore, more experiments that are specific are required. Else et al. (2015) found a relationship between the aesthetic perception of people for art and the N100; the artist group elicited a larger N100 in the frontal and central regions when watching art than the non-artist group. Guo et al. (2019) suggested that the N100 reflects the activation of specific visual features in the early visual regions of the brain and peaks earlier in the frontal and central areas than in the posterior regions. Therefore, we propose Hypothesis 1. H1: Like-tiles will induce greater N100 than dislike-tiles in the frontal and central regions.

In addition to the N100, many studies have found that ERP components within 200–400 ms are related to visual perception and emotional evaluation (Carretié et al., 1997; Höfel and Jacobsen, 2007; Yuan et al., 2007; Tommaso et al., 2008; Handy et al., 2010). The P200 (peaking around 200 ms after stimulation) reflects the early exogenous attention resources allocation (Hooff et al., 2011; Sun et al., 2015; Kosonogov et al., 2019). Some studies have shown that the P200 amplitude increases when stimuli elicit an emotional response (Carretié et al., 2004; Kanske et al., 2011). Several researchers have found that negative stimuli often elicit a greater P200 (Carretié et al., 2001; Dennis and Chen, 2007). In previous studies about visual aesthetic preferences, Wang et al. (2012) found that less beautiful pendants elicited greater P200 amplitudes than beautiful pendants. Architectural pictures with low aesthetic experience elicit greater P200 amplitudes than beautiful architectural pictures (Ma et al., 2015). Li et al. (2015) found that disliked characters elicited the highest P200. Similarly, Righi et al. (2017) found that non-preferred tools elicited greater P200 than preferred tools. According to these studies, P200 represents attention distribution, which is closely related to the emotional evaluation caused by the aesthetic perception of design. Based on previous studies on aesthetic preference in which dislike stimuli elicited greater P200, we propose Hypothesis 2. H2: Dislike-tiles produce greater P2 amplitudes than like-tiles.

The late positive potential (LPP) is a persistent component that reaches its maximum amplitude during 300–800 ms after stimulation and reflects mood assessment and sustained attention allocation (Cuthbert et al., 2000; Azizian and Polich, 2007; Schupp and Kirmse, 2021). Many studies have shown that LPP components are sensitive to the affective valence of the stimuli (Cacioppo and Berntson, 1994; Olofsson et al., 2008). Some researchers have suggested that LPP is closely related to arousal levels (Cuthbert et al., 2000; Schupp et al., 2000). Significant emotional stimuli have been shown to elicit a greater LPP than neutral emotional stimuli (Keil et al., 2002; Foti et al., 2009; Hajcak et al., 2010; Liu et al., 2019). Guo et al. (2019) suggested that LPP may indicate an assessed influence classification in preference formation in a study on the perception of people for mobile game navigation interface. Therefore, LPP is closely related to the emotional assessment of aesthetic preference. In previous research on aesthetic preference, Li et al. (2015) and Righi et al. (2017) found that the aesthetic unpleasantness of the stimuli may moderate the LPP amplitude, with dislike-stimuli eliciting greater LPP amplitudes. Wang et al. (2021) found that disliked mobile phone interfaces elicited greater LPP amplitudes. These studies led us to propose Hypothesis 3. H3: Dislike-tiles elicit greater LPP amplitudes than like-tiles.

To address our hypotheses, we posed the following research questions:

Do aesthetic preferences for tiles cause differences in ERP amplitudes (N100, P200, and LPP)?

Analyzing ERP components can reveal the relationship between human aesthetic preferences and the neural responses of ceramic tiles. This study is crucial to understanding how tiles influence human aesthetic preferences. Furthermore, it can provide designers and marketers with more information to reduce design flaws and waste, especially as the COVID-19 pandemic increases indoor office hours.

## Materials and methods

### Ethics statement

This study was conducted following the ethical guidelines of the Declaration of Helsinki. Furthermore, this study was approved by the ethics committee of the Third People’s Hospital of Jingdezhen, China (LL2022006). Each participant signed an informed consent form at the beginning of the study.

### Participants

In this study, the G*Power 3.1 software was used to calculate the minimum sample size required. A minimum sample size of 10 was required to detect a large size of 0.4 when α and power (1-β) were selected at 0.05 and 0.95, respectively. Based on previous similar studies using the ERP technique (Beudt and Jacobsen, 2015; Ma et al., 2015; Righi et al., 2017), 16 undergraduates from the Jingdezhen Ceramic Institute (eight males and eight females, 18–25 years old, mean 21.13 years old, SD = 0.661) were selected as participants. All participants completed a questionnaire related to aesthetic preference for the stimuli. Subsequently, they participated in ERP experiments. Each participant had a normal or corrected visual acuity. The following exclusion criteria were considered while recruiting participants: (1) visual impairment resulting in the inability to see visual stimuli clearly, (2) a history of neurological and psychiatric disorders, (3) diagnosis of autoimmune disease and mini mental state examination (MMSE); (4) major depressive disorder (based on DSM IV and SCL-90), and (5) presence of mild cognitive impairment (MCI). Clinicians from Jingdezhen Third People’s Hospital inspected the participants according to the criteria. In addition, all participants were asked to rest well and refrain from taking stimulants (e.g., alcohol, caffeine, and nicotine) or psychotropic drugs (e.g., steroids, immunosuppressants, and hormones) 1 week before the experiment. Each participant was reported to have rested well during the week before the experiment. They were compensated 70 CNY after the experiment.

### Stimuli

Previous research has confirmed that two-dimensional images containing tiles can be used as stimuli in neurological experiments (Laparra-Hernández et al., 2009); therefore, images were used as stimuli in the present study. After examining the characteristics of the most common ceramic tile images on the local ceramic tile market and e-commerce website https://www.taobao.com/, two researchers selected 42 ceramic tile images of different colors, brightnesses, and textures from the open-source tile model website^1^. After eliminating duplicates and blurred images, the researchers kept 40 images and invited an expert from Jingdezhen Ceramic University to evaluate the ceramic tile images. Finally, researchers and the expert agreed that the 40 ceramic tiles should be used as preliminary images. The tiles were adjusted to the same size to avoid size effects. These 40 images were used to conduct a questionnaire assessment of tiles with different levels of aesthetic preferences. Guo et al. (2022) used a 7-point Likert scale for subjects to indicate how much participants liked or disliked the appearance of an experimental stimulus in their ERP study related to preferences for robot appearance design. Ma et al. (2015) used a 7-point Likert scale to ask participants how beautiful the architectural design was. Tommaso et al. (2008) also used a 10-point Likert scale in their ERP study related to the aesthetic perception of artistic pictures by asking participants to answer how beautiful the experimental stimulus was to evaluate the level of aesthetic preference. Therefore, 16 participants were asked to assess their aesthetic preferences by answering a question. The question was: “From an aesthetic point of view, how much do you like the exterior design of this tile?” Preferences were evaluated using a 9-point Likert scale, with one representing a low preference and nine representing a high preference. At the end of the evaluation, the four highest-rated tile images were used as the like-tiles group, as shown in Table 1. The four lowest-rated tile images were used as the dislike-tiles group, with a significant difference in the mean preference scores between the two groups (like-tiles group = 6.207, dislike-tiles group = 3.775, *p* = 0.02). Images were displayed at 768 × 768 pixels on a 15.6 inch LCD monitor (60 Hz).

**TABLE 1:** Mean preference ratings of two conditions.

Tiles	Dislike-tiles		Like-tiles		T	*P*
				
	Mean	SD	Mean	SD		
Preference ratings	3.775	0.731	6.207	0.476	4.522	0.02*

*P < 0.05.

### Procedure

**FIGURE 1:**
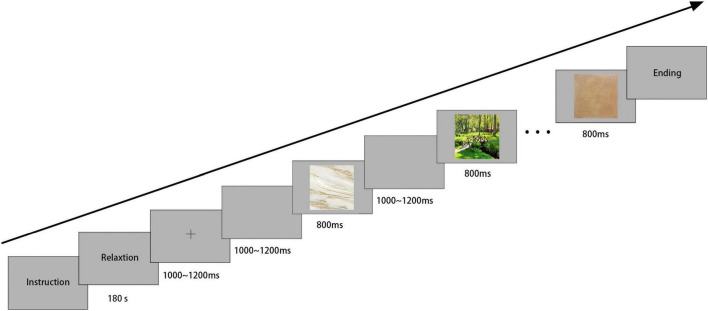
Task paradigm with a timing of presentation.

The experiment was conducted in a well-lit room. The participants sat 60 cm before a computer screen, looking at the stimuli at approximately 32.9 × 18.5 (width × height) view. This study refers to an amended oddball paradigm (Cao et al., 2021; Wan et al., 2021). The visual stimuli in the experimental task were programmed and presented using E-prime 2.0. Participants were required to view a set of images, including eight non-target tile images (320 trials) and four target landscape pictures (160 trials) for a total of 480 stimuli. A random presentation was used in the experiment to eliminate order effects. First, a 3 min countdown was shown on the screen so participants could relax. Then, a plus sign appeared in the middle of the screen to help participants focus, and the stimuli appeared at 1200 ms intervals, each lasting 800 ms until the experiment ended (Figure 1). A gray background was used for all stimuli and intervals. The intervals between stimuli were designed to help participants return to their baseline status better. The entire experiment took approximately 20 min, with one break in the middle of each experiment.

### Electrophysiological recording and analysis

**FIGURE 2:**
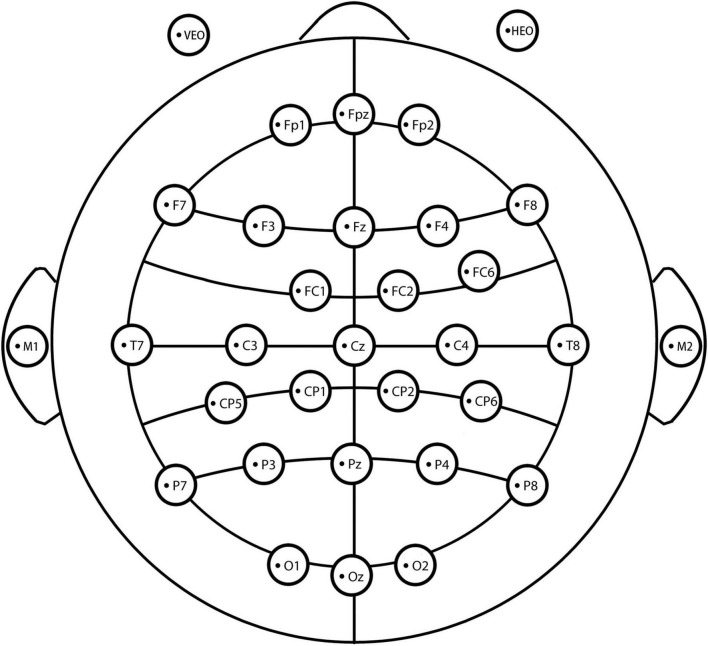
A diagram of the electrodes used in the experiment.

In this study, we used a SMARTING PRO Electroencephalogram (EEG) system with 32 electrodes to continuously record EEG signals. An extended version of the international 10–20 electrode placement system was used to place the electrodes (Figure 2). The EEG was continuously recorded from 32 standard scalp locations according to the 10–20 system (five midline electrodes: Fpz/Fz/Cz/Pz/Oz; 25 electrodes: FP1/FP2, F3/F4, F7/F8, FC1/FC2, FC6, C3/C4, T7/T8, CP1/CP2, CP5/CP6, P3/P4, P7/P8, and O1/O2; two reference electrodes: M1/M2; and two electrodes of the electro-oculogram (EOG): VEOG and HEOG). The reference electrodes were placed at the bilateral ear lobes, and the midpoint between Fpz and Fz was used as the ground electrode. In addition, vertical and horizontal EOGs were placed 1.5 cm below the left eye and the outer canthus of the right eye, respectively. During the recording, the impedances of all electrodes were reduced to less than 5 kΩ.

After recording, MATLAB2013a and EEGLAB toolboxes were used for offline analysis. The offline analysis was divided into several steps. First, the continuous EEG signal was bandpass filtered. The high-pass frequency was set to 0.1 Hz, and the low-pass frequency was set to 30 Hz. The data were then divided into single recordings from 200 ms before stimulus onset to 800 ms after stimulus onset, corrected for baseline with a mean amplitude of –200–0 ms. By visual scanning, segments with noticeable drift artifacts were removed. The earlobe potential was used as the re-reference. Independent component analysis (ICA) was performed after referencing. After removing artifacts from the ICA, segments with amplitudes greater than ±100 μv were removed. At least 30 segments per tile stimulus were available after artifact rejection. Then, the ERPs were averaged for each participant, channel, and condition. From this, the grand average ERPs were generated under two conditions: like-tiles and dislike-tiles.

Some studies related to design preference have shown that preferred stimuli induce greater N100 amplitudes in the frontal and central regions (Li et al., 2015; Guo et al., 2019; Wan et al., 2021). Therefore, the N100 amplitudes in the frontal and central regions (F3, Fz, F4, C3, Cz, and C4) were selected for the statistical analysis. Lin et al. (2018) and Dennis and Chen (2007) found that the P200 in the parietal region reflects a greater degree of automatic attentional resource allocation to negative stimuli. Wang et al. found that a low-attractiveness stimulus elicited greater P200 in the parietal region. Therefore, the P200 amplitude of the parietal region (P3, Pz, and P4) was selected for statistical analysis. Schupp et al. (2000) found that strong emotional stimulation can modulate the LPP amplitude at the Pz site. Righi et al. (2017) found that tool appearance designs with different levels of aesthetic preference induced significant LPP amplitudes in the Oz electrode. Wang et al. (2021) found that a disliked stimulus appearance elicited greater LPP in the parietal and occipital regions. Combined with the waveform of this experiment, the LPP amplitudes in the parietal and occipital regions (P3, Pz, P4, O1, Oz, and O2) were selected for the analysis. The time windows of ERPs for this study were set as follows: (1) the central region was selected within the time window of 140∼190 ms for N100, (2) the parietal region was selected within the time window of 190∼250 ms for P200, (3) the parietal and occipital regions were selected within the time window of 500∼700 ms for LPP. After determining the time windows of the components, the ERPs were averaged for specific channels and experimental conditions within each time window.

The mean amplitudes of each channel within each time window were entered into multi-factor repeated-measures ANOVAs (N100, P200, and LPP). Each ANOVA included two factors: preference (dislike-tiles, like-tiles) and electrode position. The analyzed data were corrected using Greenhouse-Geisser. All statistical analyses were tested for statistical significance (α = 0.05) using IBM SPSS Statistics 25.

## Results

Brain activity signals of the participants evoked by visual stimuli were recorded and analyzed. The data were analyzed using event-related potential theory and techniques. The analysis results of the N100, P200, and LPP components are as follows.

Repeated-measures ANOVA revealed that participant preference for tiles significantly affected the N100 (140–190 ms) amplitudes in the frontal and central regions [F (1, 15) = 7.799, *P* = 0.014, partial η^2^ = 0.342]. There was no significant effect on electrode position [F (1, 15) = 1.826, *P* = 0.183, partial η^2^ = 0.109] and no significant interaction between preference and electrodes [F (1, 15) = 1.595, *P* = 0.206, partial η^2^ = 0.096]. The mean N100 amplitude induced by the like-tiles (mean = –1.102, SD = 0.61) was lower than that induced by the dislike-tiles (mean = –0.488, SD = 0.615).

For P200 (190–250 ms), repeated measures ANOVA revealed a significant effect of preference on the P200 amplitudes of the parietal region [F (1, 15) = 5.072, *P* = 0.04, partial η^2^ = 0.253]. There was no significant effect of electrode position [F (1, 15) = 3.215, *P* = 0.084, partial η^2^ = 0.177] and no significant interaction between preference and electrodes [F (1, 15) = 1.402, *P* = 0.257, partial η^2^ = 0.085]. The mean P200 amplitude induced by the dislike-tiles (mean = 1.855, SD = 0.84) was higher than that induced by the like-tiles (mean = 1.254, SD = 0.84).

Repeated-measures ANOVA results revealed a significant effect of preference on LPP (500–700 ms) amplitudes in the parietal and occipital regions [F (1, 15) = 5.204, *P* = 0.038, partial η^2^ = 0.258]. There was no significant effect on electrode position [F (1, 15) = 2.458, *P* = 0.089, partial η^2^ = 0.141] and no significant interaction between preference and electrodes [F (1, 15) = 0.446, *P* = 0.707, partial η^2^ = 0.029]. The mean LPP amplitude induced by the dislike-tiles (mean = 2.072, SD = 1.28) was higher than that induced by the like-tiles (mean = 1.322, SD = 1.176).

**FIGURE 3:**
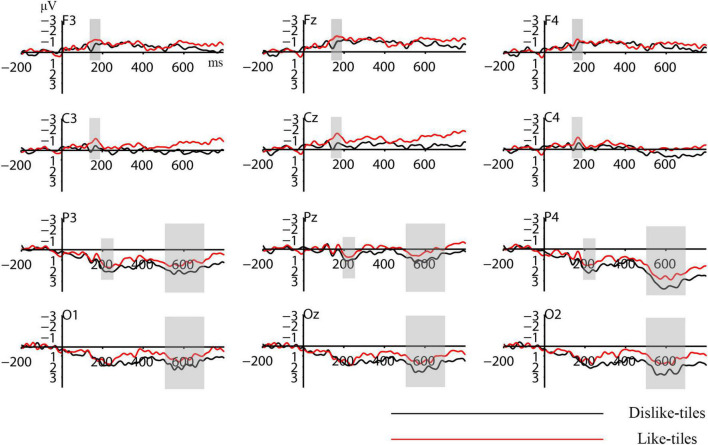
The grand averaged waveforms of each electrode under the two conditions. The x-axis indicates the time, and the y-axis indicates the voltage.

**FIGURE 4:**
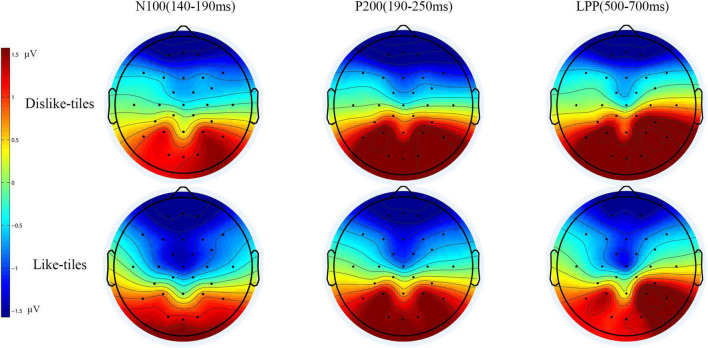
The scalp topography under the two conditions.

Figure 3 shows the waveforms of each electrode under these two conditions. Figure 4 shows the brain topography of the N100, P200, and LPP. Table 2 lists the differences in the mean amplitudes between the two conditions.

**TABLE 2:** Mean amplitudes (μV) of each region under two conditions.

ERPs	Locations	Dislike-tiles		Like-tiles		F	*P*	partial η^2^
						
		Mean	SD	Mean	SD			
N100	Frontal region (F3/Fz/F4) Central region (C3/Cz/C4)	–0.488	0.615	–1.102	0.61	7.799	0.014*	0.342
P200	Parietal region (P3/Pz/P4)	1.855	0.84	1.254	0.84	5.072	0.04*	0.253
LPP	Parietal region (P3/Pz/P4) Occipital region (O1/Oz/O2)	2.072	1.28	1.322	1.176	5.204	0.038*	0.258

*P < 0.05.

## Discussion

This study explored the relationship between preferences for tile appearance and neural responses. We specifically recorded the EEG signals of the participants evoked by different tiles and analyzed the ERPs. The results showed that aesthetic preference had a powerful modulatory effect on the underlying emotional and cognitive processes of brain activity. Furthermore, the preference factors caused significant differences in the amplitudes of N100, P200, and LPP.

### N100

The visual stimulus-triggered N100 reflects automatic perceptual processing and attentional resource allocation (Vogel and Luck, 2000). Previous neuroaesthetic studies have found that the processing of brightness, color, and grouping in the early stages of visual aesthetics occurs in relevant parts of the occipital region. In contrast, several regions of the emotional evaluation system in the brain, such as the medial frontal cortex, contribute to aesthetic experience (Chatterjee and Vartanian, 2014). Our stimulus-triggered N100 component was significant in the frontal and central regions, nevertheless not in the occipital region. Therefore, the N100 component in this study may not be caused by visual differences, such as color; rather, it is related to aesthetic experience. However, the possibility of a relationship between the N100 amplitude and visual feature differences between the two groups of tiles cannot be completely excluded. This point is one of the limitations of this study and will be explored further in future studies. Many researchers believe that the attractiveness of the physical feelings in people, and attributes of the stimuli trigger appreciation and emotions of pleasant or unpleasant, influencing preference evaluation (Handy et al., 2010; Kawasaki and Yamaguchi, 2012). Keil et al. (2002) found that pleasant and unpleasant emotional stimuli elicited a greater N100 than neutral emotional stimuli. Thus, the N100 can be used to reveal the allocation of attention to preference formation (Guo et al., 2018; Liu et al., 2021). The fact that disliked stimuli elicited greater N100 in the study (Liu et al., 2019) may be because disliked stimuli elicited more unpleasant emotions attracting more attention, which can be attributed to the fact that the experimental stimuli they used were different from those in this study. In addition, Else et al. (2015) found that viewing art images elicited greater N100 in frontal and central regions in the artist group than in the non-artist group. The artist group had stronger attention and aesthetic perception ability toward artworks than the non-artist group, indicating that N100 has a specific relationship with aesthetically related emotions. According to previous theory (Chatterjee and Vartanian, 2014) and interpretation (Else et al., 2015; Guo et al., 2018; Liu et al., 2021), like-tiles evoked greater N100 in the prefrontal and central regions than the dislike-tiles in this study, possibly because the like-tiles evoked positive emotions and attracted more attention. These findings support the idea that aesthetic preferences of people moderate the amplitude of N100 components, and like-stimuli can elicit a greater N100 than dislike-stimuli, which is consistent with previous findings (Wang et al., 2012; Liu et al., 2021; Guo et al., 2022). The results of this study support hypothesis 1.

### P200

The P200 is the ERP component associated with visual assessment within 200–400 ms after stimulus onset (Höfel and Jacobsen, 2007; Tommaso et al., 2008). The P200 in this study was sensitive to preference factors, from which it can be inferred that perceptual detection of preference by the P200 component is a bottom-up process driven by the stimulus. Previous studies have demonstrated that the P200 component is related to visual attention (Hooff et al., 2011; Sun et al., 2015; Kosonogov et al., 2019). Therefore, the P200 in the parietal region reflects a greater degree of automatic attentional resource allocation to negative stimuli (Dennis and Chen, 2007; Lin et al., 2018). According to this interpretation, the results of this study may be due to dislike-tiles triggering more negative emotions, attracting the attention of people automatically. The present results support the idea that dislike-stimuli may elicit significant negative emotions, thus, attracting more attention and eliciting higher P200 amplitudes than like-stimuli. Furthermore, although the experimental stimuli in this study were different from those of previous studies, the present findings are consistent with those of previous studies (Wang et al., 2012; Li et al., 2015; Ma et al., 2015; Righi et al., 2017). Therefore, aesthetic preference had a moderating effect on P200 amplitude, with dislike-stimuli eliciting greater P200 amplitudes. This result supports hypothesis 2.

### Late positive potential

The LPP is thought to reflect emotional assessment and sustained attention allocation (Hajcak and Nieuwenhuis, 2006; Sabatinelli et al., 2007; Foti et al., 2009; Hajcak et al., 2009). Within 500–700 ms of stimulation, we considered the LPP component. The LPP component is closely related to the later emotional assessment of experimental stimuli (Cuthbert et al., 2000; Azizian and Polich, 2007). Many studies have shown that the LPP is sensitive to strong emotional stimuli (Olofsson et al., 2008; Handy et al., 2010). Several studies also demonstrate that LPP amplitude positively correlates with attention levels (Fox, 1994; Ito and Cacioppo, 2000; Mothes-Lasch et al., 2013). The amplitude of the LPP decreases with decreasing attention levels (Pessoa et al., 2002; Bishop et al., 2007). The LPP amplitude increases when attention is shifted to the emotional features of the stimulus (Hajcak et al., 2006; Schindler and Straube, 2020). The dislike-tiles in the present study triggered greater LPP amplitudes. One interpretation of the affective effect of LPP is that people have a negative bias (i.e., people prioritize unpleasant stimuli over pleasant ones) (Ito et al., 1998). Based on this interpretation, this may be because dislike-tiles were more likely to elicit negative affect in participants, attracting more attention. Our findings confirm that preference factors moderate LPP amplitude and that dislike-stimuli trigger greater LPP (Li et al., 2015; Ma et al., 2015; Righi et al., 2017; Wang et al., 2021). The results of this study suggest that implicit emotion regulation is induced by aesthetic preference, and that attention to stimuli with lower aesthetic attributes may play a role. This result supports hypothesis 3.

## Conclusion

The results of this study support the idea that aesthetic preferences for tiles can modulate the underlying emotional and cognitive processes of brain activity. Aesthetic preferences for product design have been the focus of designers, marketers, and companies. This study was conducted by recording ERPs of participants in low- and high-aesthetic preference tile conditions. Specifically, the hypotheses were tested by showing differences in ERP waveforms between the two groups with different visual stimuli. The results showed that people elicited greater N100 amplitudes for the like-tiles, possibly indicating that attentional resources were allocated more to the like-tiles at first and elicited greater P200 and LPP amplitudes indicating that the dislike-tiles evoked more negative emotions than like-tiles.

From a theoretical perspective, the results of this study can help designers and marketers better understand aesthetic preferences for tile design. According to the visual processing stage result, people have already formed a preliminary impression of ceramic tiles in the early and middle stages of vision. They have formed different allocations of attention to ceramic tiles with different aesthetic preference levels. This is reflected in the main effect of aesthetic preference on N100 and P200 amplitudes, with like-tiles eliciting a greater N100, indicating that like-tiles were noticed first. The dislike-tiles elicit greater P200, indicating that people pay more attention to the dislike-tile during this period. Finally, the greater LPP amplitude triggered by the dislike-tiles indicates that the impression of people toward the tiles deepened further in the late stage, resulting in top-down emotion-driven evaluation. Overall, the dislike-tiles induced greater emotional responses and attention allocation than the like-tiles did. These findings contribute to the development of neuroaesthetic research and provide several results and indices for tiles. Future research needs to explore different interior design colors, including tile colors, wall surfaces, and interior elements (e.g., furniture). The method provided in this paper, through ERP technology, expands the research perspective of tile design and environmental design. From a practical perspective, this study offers instructions for interior designers, environmental designers, and other interested parties. Evaluating the aesthetic quality of ceramic tiles is helpful for designers to carry out environmental design better and improve their living environment.

## Limitation

First, although our number of participants reached the minimum required for statistical analysis, it should be as large as possible. Therefore, we will increase the number of participants in future studies. Second, most participants in this study were young people between the ages of 18 and 25 years, requiring the recruitment of multiple age groups in future studies. In addition, the experience of people with tiles in shopping is also influenced by factors such as price, which will be studied further in future research. Finally, the preferences of people for ceramic tiles were determined using a questionnaire before the ERP experiment in this study. However, collecting the behavioral responses of the participants in the ERP experiment may intuitively reflect the preference for tiles. Hence, the behavioral responses of participants will be included in future ERP experiments.

## Data availability statement

The original contributions presented in the study are included in the article/Supplementary material, further inquiries can be directed to the corresponding author.

## Ethics statement

The studies involving human participants were reviewed and approved by Jingdezhen Third People’s Hospital, China. The patients/participants provided their written informed consent to participate in this study.

## Author contributions

JC was involved in study design, execution, data analysis, manuscript drafting, and revision. YC was involved in manuscript revision. Both authors contributed to the article and approved the submitted version.

## Footnotes

1
https://www.om.cn/models/pic-408-541-0-0-0-2-0-0-1-0-0-0-0-0.html
